# Needle acquisition patterns, network risk and social capital among rural PWID in Puerto Rico

**DOI:** 10.1186/s12954-017-0195-5

**Published:** 2017-10-18

**Authors:** Ian Duncan, Patrick Habecker, Roberto Abadie, Ric Curtis, Bilal Khan, Kirk Dombrowski

**Affiliations:** 1 University of Nebraska, Lincoln, Nebraska USA; 2 John Jay College of Criminal Justice, New York, USA

## Abstract

**Background:**

People who inject drugs (PWID) take on significant risks of contracting blood-borne infection, including injecting with a large number of partners and acquiring needles from unsafe sources. When combined, risk of infection can be magnified.

**Methods:**

Using a sample of PWID in rural Puerto Rico, we model the relationship between a subject’s number of injection partners and the likelihood of having used an unsafe source of injection syringes. Data collection with 315 current injectors identified six sources of needles.

**Results:**

Of the six possible sources, only acquisition from a seller (paid or free), or using syringes found on the street, was significantly related to number of partners.

**Conclusions:**

These results suggest that sources of syringes do serve to multiply risk of infection caused by multi-partner injection concurrency. They also suggest that prior research on distinct forms of social capital among PWID may need to be rethought.

## Background

It is often taken for granted that people who inject drugs take on significant risks of infection. In addition to the potentially harmful effects that injecting drugs can have in and of itself, there is also the additional concern of spreading disease through the sharing of needles or other equipment—such as syringes, cookers, or cotton used to filter the solution before it is drawn into the syringe. Despite these risks, people who inject drugs (PWID) often share injection equipment, both as a cost-cutting measure [1] and as a way to establish and maintain relationships [2]. This risk behavior has received considerable attention in the past—resulting in early and consistent efforts by syringe exchange and syringe service programs since the 1980s [3]. The success of such harm reduction programs is clear [4], but recent work has shown a dearth of services in rural areas [5]. The aim of this paper is to examine how, for rural PWID, the number of risk partners in an individual’s injection network influences or is influenced by where they obtained their injection syringes.

Understanding the unique risks associated with rural injection is significant; the last 10 years have shown a dramatic rise in rural injection drug use [6, 7]. Rural syringe access programs face large challenges, most especially the difficulty they face in reaching a highly dispersed population [8]. Under these circumstances, attention to the ongoing risk posed by risky syringe access is critical.

There are a number of sources from which both rural and urban PWID acquire syringes, such as syringe exchange programs, pharmacies, or the secondary market (e.g., drug sellers, other injectors, etc.). Each of these sources comes with its own unique constellation of risks based on how likely the syringe received is to be sterile—from certainly sterile syringes obtained via pharmacies and exchanges that carry no risk to highly risky, potentially contaminated syringes found on the street. Not all PWID have equal access to these sources. Social constraints influence syringe access in many rural areas, especially where formal syringe exchange programs remain illegal [9]. Where rural pharmacies have the potential to fill this gap, research has shown considerable variability in the receptiveness of pharmacies to serve the PWID population [10]. Under such conditions, syringe access sources do not necessarily reflect choices by drug users, at least in any strict sense. Rather, information on syringe access also (and perhaps only) points to ways in which access to sterile injection equipment may be decided by larger structural factors, such as syringe availability and the presence or absence of individual social capital [11]. In this sense, a focus on syringe sources may best be understood as a study of the extent to which the structural issues that govern users lives potentially limit their opportunities to engage in safe injection practices, putting the larger community of drug users (and the community in which those users are embedded) at risk of infection [12].

The range of strategies for obtaining injection-related syringes is large, though most of our information on the topic comes from research in urban areas. In large US cities, buying a syringe on the secondary market is a common means of acquisition [4, 13]. For example, Latkin and Forman [14] examined PWID in Baltimore, finding that designated sellers were, by far, the most common source of syringes for their sample. This is a worrisome finding, as participants who sold needles and syringes reported that it was easy to make used equipment appear new, and some admitted to reselling re-selling used syringes, while others implied this practice was common though they did not do it. In all, only 8% of respondents in this study reported using exclusively safe sources [14]. These were predominately from the formal market (exchanges, pharmacies, and hospitals; we note that secondary exchange with an insulin-injecting diabetic was considered a safe source in their research).

Other urban studies have found that access to exchange programs or other methods of generally safer access, such as pharmacies, decreased needle and syringe sharing among PWID [15–17]. Research by Fuller and colleagues [18] found that needle/syringe access programs did not lead to more unsafely discarded syringes in the neighborhood, as some opponents worried. However, research from Iran found that insufficient access to an exchange program was associated with more needle and syringe re-use [19], and similar research in US cities found that use of exchange programs was associated with decreased equipment sharing overall, though the relationship was fairly weak [20].

Craine and colleagues [21] compared respondents’ patterns of pharmacy-based purchases, non-pharmacy-based exchanges, or use of the secondary market (e.g., street sellers), and found distinctive user profiles based on source frequencies. For example, PWID who typically used non-pharmacy exchanges had higher injection frequencies and were more likely to be homeless, whereas those who typically used the secondary market were comparatively younger and more recent initiators of injection drug use than their peers. These findings suggest that, while exchange programs are beneficial, they do not necessarily reach across all user groups, leaving distinct sets of drug users with much higher risk profiles than others.

The impact of rurality on the use of safe injection equipment has received less attention. Recent work by our study team found that, in both rural and urban Puerto Rico, utilizing syringe exchange programs significantly increases the frequency of using sterile equipment to inject [8] and that rural users faced significantly lower access to exchange programs. Other research on rural drug use suggests that syringe sharing remains common, despite the prevalence of safer options [22].

Despite the above findings, basic demographic variables have shown mixed results as predictors of syringe access patterns. For example, studies examining the role of gender on syringe acquisition show more risky injection behaviors among women, such as needle borrowing [23] and equipment sharing in general [24], but also finding that women are less likely to use the secondary market as a source of syringes [25]. Age has been identified as a generally reliable factor for syringe source risk, concluding that younger injectors exhibiting more injection-related risks than older injectors [26–28]. This includes research using an urban Australian sample that found that among young people transitioning to injection, there was little knowledge about sources of safe injection equipment [29]. For LGB respondents, however, it was those without stable housing who were more likely to use the secondary market of friends and sellers [30]. Craine et al. [21] found just the opposite—that homelessness was positively related to preferring the formal market, such as non-pharmacy exchanges, and avoiding riskier sources. Furthermore, some research finds that, when choice is possible, the preferred source is related to individual tendencies to share equipment [31], whereas others find no such relationship [30]. While PWID in most studies are predominately low-income [32], income differential has been shown to affect syringe-related risk behaviors: higher-income PWID are more likely to take protective measures when they inject, such as only selecting partners with similar viral statuses [33]. Conversely, research in Iran found that homeless participants were more likely than those with stable housing to access needles on the secondary market [34].

While the demographics of users have been shown to hold some predictive power in understanding structural factors that affect injection risk, it is important to consider an additional potential predictor: risk network size. Prior studies have analyzed the risk of sharing syringes in networks, but few have examined syringe acquisition behavior in relation to overall co-injector network degree [34]. More common are studies that examine social support in regulating sharing [35] or network topological factors that influence transmission of infection across whole networks [36–39]. While some have examined the link between personal network size and syringe sharing behaviors [40], as far as we know, no one has looked at the relationship between personal network size and syringe access.

In what follows, we treat personal network size as a risk behavior [41, 42]. By examining the number of users with whom one has shared syringes, we make use of a quantitative measure that is closely tied to both individual risk of infection and overall network topology [43, 44]. If a positive association is found between network size and use of unsafe sources for syringes, the system-wide importance of individual acts rises significantly [38, 45]. For these reasons, examining the relationship between co-injection network size and a range of syringe acquisition sources is an important next step in understanding structural and personal network factors related to injection risk.

In the analysis below, we test the hypothesis that those who inject with more partners will be more likely to depend on syringes from higher risk sources. There are reasons to believe such a relationship will exist. Both of these factors can be seen to involve questions of limited resources, venue-based limited opportunities (as when drug use takes place in a shooting gallery), and a reliance on long-term social relationships. In addition, ethnographic fieldwork at our research locale pointed to important social dynamics that influence syringe needs and the relationships that can meet those needs. Mixed qualitative and quantitative analyses have shown that in rural Puerto Rico, co-injection network size is correlated with frequency of drug use—an outgrowth of the fact that frequent users require more opportunities for collaborating in a local injection practice known as *caballo* [46]. In rural Puerto Rico, *caballo* involves two PWID pooling financial resources to obtain both heroin and cocaine for mixed injection (known in the drug literature as a “speedball”), with the two *caballo* partners then sharing the combined drugs. This is a favored local form of injection, particularly among frequent users in rural Puerto Rico where it will sometimes be performed ten or more times each day. By pooling resources, users can get ½ bag of heroin and ½ bag of cocaine for roughly what it would cost them for a whole bag of one or the other.

The main use of a low-dose speedball in this way is stave off withdrawal while counteracting the soporific effects of the heroin, leaving the user sufficiently functional to go about the task of daily life. Frequent injectors tend to maximize the size of their co-injection network to allow greater opportunities for pooling/sharing of resources. Yet, higher rates of injection also imply the need for a higher number of syringes. Given the limited time available between uses prior to experiencing withdrawal, such practices suggest that those with high frequency of use and high numbers of partners would more regularly resort to less safe sources for injection equipment. This logic is supported by ethnographic evidence from our study which noted that high-frequency users were likely to rely on exchange programs for some of their syringes, but that the high number required for multiple daily use (often exceeding 10 times per day) meant that the number of syringes received from SEPs will not be enough for their needs. Re-using or buying from peers or from drug sellers was a common strategy to make up the difference. Such strategies, however, magnify overall risk of infection, coupling the number of concurrent partners with increasing use of unsafe injection equipment. Our hypothesis is meant to reflect this possibility. We make this prediction with caution, however, as previous analyses found that PWID behaviors are strongly influenced by local norms governing injection equipment sharing and overall injection practices [47]. In addition, we utilize a sample of rural Puerto Rican PWID, and there is evidence that urban Puerto Rican injectors have higher rates of syringe sharing more generally [48] and on average inject more frequently [49] than PWID on the US mainland. Furthermore, rural PWID are less influenced by infection status of potential co-users when selecting co-injection partners [50].

### Data

Interviews with 315 participants were completed between April 19 and June 15, 2015, in a rural area approximately 30 miles from San Juan, Puerto Rico, drawing participants from several surrounding towns. Eligible participants were alert, 18 years of age or older, and reported injecting drugs within the last 30 days. Visual inspections for injection signs as well as questionnaires about drug injection knowledge were used to confirm this. Upon completing the questionnaire, participants were compensated with $25. Recruitment into the sample was managed using Respondent-Driven Sampling (RDS) whereby participants who completed the survey were given three referral coupons that they could pass out to other qualified individuals who had not previously participated in the project. Recruitment began with eight total seeds, two from each community in the area. For every referral that completed the survey, the referee could earn an additional $10. RDS is often the preferred method for recruiting stigmatized populations and has a strong track record for reliable results when used to produce samples of PWID [51–53]. Seeds were identified through a local, mobile, weekly syringe exchange program. Though there are concerns about representativeness using this method of recruitment, analyses of the sampling bias show low levels of demographic or injection behavior homophily and minimal degree effects in the recruitment process [54, 55]. Analysis of referral data showed that past participation in drug treatment had more of an effect on network ties than other factors, and use/non-use of syringe exchange did not seem to have a marked influence on recruitment patterns.

The questionnaire itself was interviewer-administered and based on the CDC National HIV Behavioral Surveillance (NHBS) IDU (injection drug use) Round 3 Questionnaire, version 3. The instrument asked questions about injection behavior, hepatitis C (HCV) and HIV status and testing histories, and several other topics related to drug use and HIV/HCV risk. In addition to recording the participants’ self-reported HCV and HIV status prior to participating in the study, the project provided rapid testing for both HIV and HCV using INSTI Rapid HIV antibody tests (Biolytical Laboratories) and OraQuick HCV Rapid antibody tests (OraSure Technologies). Participants were compensated an additional $5 for each test completed. Participants who tested positive for HCV or HIV were offered referral and transportation to a primary care doctor for confirmatory testing and potential treatment. The study received IRB approval through the University of Nebraska-Lincoln (IRB# 20131113844FB) and the University of Puerto Rico School of Medicine (IRB# A8480115).

## Methods

We use six distinct dependent variables, each corresponding to whether or not the participant had acquired a needle from a specific source over the past year. These six sources are (1) a syringe exchange program; (2) a local pharmacy; (3) free from a friend, acquaintance, relative, or sex partner; (4) free from someone else, such as their dealer or gallery operator; (5) paid for a syringe from a dealer, shooting gallery operator, or other vendors on the street; and (6) found a syringe on the street. These questions were asked independently, and respondents answered yes or no. Exchange programs and pharmacies can be thought of as the formal market, whereas the other sources reside on the secondary market. Unfortunately, the data includes no information on the number of syringes obtained in the past year from each location, just if the source was used or not. In the survey, users were allowed to answer yes or no for each category but were not asked to rank the list of possible sources. For this reason, each source is analyzed individually.

Our *primary independent variable* is the number of partners a respondent had co-injected with and used the syringe after their partner did, over the past year. This is a highly restrictive notion of injection partner—one with whom a respondent has participated in an injection behavior that risks —that is meant to ensure we are assessing actual infection risk when using the measure as a statistical predictor of accessing syringes from a particular source. Respondents were asked, “In the last 12 months, with how many people did you use a needle after they injected with it?” This is an important distinction from simply number of *caballo* or other risk partners, as the risk of viral infection is inherently larger for people who inject second.

Several control variables were used in this analysis. These included (1) marital/co-habitation status (1 = married/cohabitating, 0 = single/other); (2) if participant annual income was $5000 or more (yes = 1); (3) gender (1 = male, 0 = female); (4) age; (5) age at first injection; (6) homeless in the past year (yes = 1); (7) and ordinal measure of injection frequency (1 = 1–3 times/month, 2 = 1–6 times/week, 3 = 1–3 times/day, 4 = 4+ times/day); (8) HCV status (dummy variable, with “unknown” as the omitted category); and (9) HIV status (dummy variable, with “unknown” as the omitted category).

We ran six models, one for each syringe source. In the model with “found needle on the street” as the source, gender and HCV status were removed from the model because they contain zero counts for each of these cells. A number of the initial controls were found to be insignificant, and additional condensed models were run for each source without them. Both sets of regression analyses are reported below. Though our total sample included 315 respondents, our analytical sample here is smaller (*n* = 245). Cases were removed due to missing data on at least one measure used across the models. Descriptive statistics of the analytic sample can be found in Table 1.

**Table 1:** Descriptive statistics

Variables	Percent/mean	Standard dev	Min	Max
Syringe Sources, past year:				
From exchange	56.33%		0	1
From pharmacy	32.24%		0	1
From friends/family	32.65%		0	1
From seller (paid)	54.29%		0	1
From seller (free)	13.06%		0	1
From the street	4.05%		0	1
Total number of co-injection partners, past year (binned at 15+)	0.857	2.006	0	15
Married/living with partner	23.67%		0	1
Annual income of $5000+	21.22%		0	1
Male	89.39%		0	1
Current age (binned at 70+)	41.873	10.162	18	70
Age at first injection	22.082	8.331	11	58
Homeless, past year	38.54%		0	1
Injection frequency	3.184	0.822	1	4
1–3 times a month (1)	5.71%			
1–6 times a week (2)	8.98%			
1–3 times a day (3)	46.53%			
4+ times a day (4)	38.78%			
HCV status				
Positive	52.24%		0	1
Negative	26.53%		0	1
Unknown	21.22%		0	1
HIV status				
Positive	4.08%		0	1
Negative	83.27%		0	1
Unknown	12.65%		0	1

## Results

Among the six syringe acquisition sources examined (Tables 2, 3, 4, 5, 6 and 7), only the three riskiest were significantly associated with number of injection partners in the full model: using a syringe found on the street (Table 7), buying a syringe from a seller (Table 6), and receiving a free syringe from a seller (Table 5). For each additional injection partner, a respondent had, their odds of having using a syringe found on the street increased by a factor of 1.282 (CI 1.051, 1.563). For each additional partner, the odds of having bought a syringe from a seller in the past year increased by a factor of 1.260 (CI 1.022, 1.552) compared to having not bought from their sellers. Finally, for each additional co-injection partner, the odds of having used a free needle from a seller increased by a factor of 1.346 (CI 1.110, 1.632). Among our control variables, none were consistently associated across all six possible options, though several had a significant relationship with one or more needle source. These relationships include:

- Higher injection frequencies (though not higher number of risk partners) were associated with having used a needle exchange program in the past year, as was having a HCV+ status (Table 2). For each unit increase on the 4-level injection frequency scale used, respondents were 1.688 (CI 1.197, 2.381) times as likely to have used a needle exchange in the past year. Respondents who had contracted the hepatitis C virus were 2.128 (CI 1.032, 4.385) times as likely to have used an exchange in the past year compared to their peers without HCV or who’s HCV status was unknown.
- Neither our main independent variable nor any of our controls were found to be significant predictors for using a pharmacy as a source (Table 3). This was the only source that had no significant predictors.
- Obtaining needles through friends and relatives was only associated with current age (Table 4). For each additional year, the odds of a respondent having used a syringe obtained from a friend or family member decreased by a factor of 0.967 (CI 0.937, 0.998).
- In addition to our main independent variable, respondents who were homeless during the past year were more likely to have used a free syringe from a seller. Conversely, those with a confirmed HCV− status were less likely, as were respondents with a confirmed HIV+ status. However, these were only marginally significant relationships.
- Finally, no control variables were significant predictors for either using a syringe bought from a seller or using one found on the street.

**Table 2:** Two-tailed binary logistic regression on needle source: exchange

	OR (full)	CI	***	OR (condensed)	CI	***
Number of injection partners	0.998	0.872, 0.142		0.989	0.869, 1.126	
Married/cohab	0.892	0.467, 1.705				
Income over $5000	1.285	0.647, 2.552				
Male	0.911	0.369, 2.247				
Age now	1.010	0.980, 1.040				
Age, first injection	1.003	0.968, 1.039				
Homeless, past year	0.923	0.513, 1.661				
Injection frequency	1.688	1.197, 2.381	**	1.577	1.140, 2.182	**
HCV status+	2.128	1.032, 4.385	*	2.073	1.057, 4.064	*
HCV status−	2.117	0.936, 4.785		1.935	0.905, 4.134	
HIV status+	2.314	0.353, 15.175				
HIV status−	0.547	0.227, 1.198				

*N* = 245

*P* values: *** ≥ 0.001; ** ≥ 0.01; * ≥ 0.05; + ≥ 0.1

**Table 3:** Two-tailed binary logistic regression on needle source: pharmacy

	OR (full)	CI	***	OR (condensed)	CI	***
Number of injection partners	0.949	0.813, 1.108		0.941	0.809, 1.095	
Married/cohab	0.899	0.457, 1.770				
Income over $5000	1.038	0.513, 2.100				
Male	0.686	0.283, 1.664				
Age now	1.021	0.990, 1.052				
Age, first injection	0.985	0.950, 1.021				
Homeless, past year	0.946	0.514, 1.739				
Injection frequency	1.058	0.751, 1.491				
HCV status+	0.708	0.337, 1.490				
HCV status−	1.146	0.505, 2.603				
HIV Status+	1.555	0.311, 7.770				
HIV Status−	1.082	0.442, 2.648				

*N* = 245

*P* values: *** ≥ 0.001; ** ≥ 0.01; * ≥ 0.05; + ≥ 0.1

**Table 4:** Two-tailed binary logistic regression on needle source: friend or relative

	OR (full)	CI	***	OR (condensed)	CI	***
Number of injection partners	1.008	0.876, 1.160		1.006	0.879, 1.151	
Married/cohab	0.843	0.426, 1.670				
Income over $5000	1.908	0.948, 3.841				
Male	0.544	0.224, 1.318				
Age now	0.967	0.937, 0.998	*	0.967	0.941, 0.994	*
Age, first injection	1.014	0.977, 1.053				
Homeless, past year	1.422	0.762, 2.651				
Injection frequency	0.838	0.592, 1.187				
HCV Status+	0.743	0.352, 1.568				
HCV Status−	0.861	0.372, 1.991				
HIV Status+	0.521	0.082, 3.319				
HIV Status−	0.803	0.335, 1.925				

*N* = 245

*P* values: *** ≥ 0.001; ** ≥ 0.01; * ≥ 0.05; + ≥ 0.1

**Table 5:** Two-tailed binary logistic regression on needle source: seller, free

	OR (full)	CI	***	OR (condensed)	CI	***
Number of injection partners	1.346	1.110, 1.632	**	1.346	1.104, 1.1642	**
Married/cohab	0.567	0.178, 1.804				
Income over $5000	1.909	0.630, 5.782				
Male	1.429	0.284, 7.197				
Age now	0.963	0.920, 1.009				
Age, first injection	0.986	0.911, 1.068				
Homeless, past year	2.253	0.893, 5.686	+	1.934	0.844, 4.429	
Injection frequency	1.363	0.772, 2.404				
HCV status+	0.811	0.291, 2.262		0.663	0.257, 1.712	
HCV status−	0.262	0.060, 1.150	+	0.242	0.057, 1.022	
HIV status+	0.694	0.061, 7.851	+	0.378	0.123, 0.927	
HIV Status−	0.397	0.134, 1.175		0.338	0.110, 0.804	

*N* = 245

*P*-values: *** ≥ 0.001; ** ≥ 0.01; * ≥ 0.05; + ≥ 0.1

**Table 6:** Two-tailed binary logistic regression on needle source: seller, paid

	OR (full)	CI	***	OR (condensed)	CI	***
Number of Injection Partners	1.260	1.022, 1.552	*	1.308	1.068, 1.600	**
Married/cohab	0.767	0.400, 1.468				
Income over $5000	1.251	0.634, 2.470				
Male	1.440	0.598, 3.468				
Age now	0.993	0.964, 1.023				
Age, first injection	0.977	0.942, 1.012				
Homeless, past year	1.443	0.801, 2.600				
Injection frequency	0.932	0.671, 1.296				
HCV status+	1.241	0.609, 2.529				
HCV Status−	1.214	0.541, 2.723				
HIV Status+	0.280	0.046, 1.712				
HIV Status−	1.473	0.633, 3.426				

*N* = 245

*P* values: *** ≥ 0.001; ** ≥ 0.01; * ≥ 0.05; + ≥ 0.1

**Table 7:** Two-tailed binary logistic regression on needle source: street

	OR (full)	CI	***	OR (condensed)	CI	***
Number of injection partners	1.282	1.051, 1.563	*	1.289	1.092, 1.520	**
Married/cohab	0.374	0.040, 3.507				
Income over $5000	0.618	0.069, 5.558				
Age now	0.999	0.933, 1.070				
Age, first injection	0.873	0.727, 1.049				
Homeless, past year	0.772	0.175, 3.398				
Injection frequency	0.775	0.341, 1.763				
HIV Status+	1.696	0.098, 29.391				
HIV Status−	0.447	0.078, 2.547				

*N* = 245

*P* values: *** ≥ 0.001; ** ≥ 0.01; * ≥ 0.05; + ≥ 0.1

Additional condensed models were conducted for each source using only the variables that were significant or marginally significant predictors in the full model. In all but one case, the results were the same. The lone exception was the condensed model for using a syringe given to you for free from a seller. None of the marginally significant predictors from the full model retained any predictive power in the condensed model, suggesting a confounding effect.

## Discussion

Of the six potential sources, only the three riskiest were associated with having a larger co-injection network size. These included (1) buying a syringe from a seller, (2) using a free syringe from a seller, and (3) using a syringe found on the street. These findings raise questions about the social structural factors that condition syringe access. Foremost among these is the question of the relationship between co-injectors: presumably, PWID with larger co-injection networks would have more opportunities to acquire syringes from friends who they inject with, yet we found no evidence of such a relationship. In considering the importance of this finding, it is important to recognize that, while syringe sharing behaviors are often conceived of as involving decisions or choices by individuals, user decisions are conditioned by the range of syringe access opportunities and their limits. One can only make the decision to use a sterile equipment if one has access to it. In the absence of a full range of choices, what may appear like modifiable behaviors may not be nearly so malleable. Rather than reading the results of this analysis as a reflection on individual choices, decisions, or behaviors [56, 57]—an interpretation that would be possible only where everyone has equal access to all possible sources—we instead consider these results as a reflection of the social capital of rural PWID as it is expressed in their injection behaviors, including syringe acquisition [11, 29].

Such a finding points to an important distinction in risk-related social determinants of health: that forms of social capital associated with drug access do not necessarily reflect greater social capacity for risk reduction. Social connections have been implicated as an important source of social capital among PWID [58–61], including recent research that has drawn attention to what has been referred to (unfortunately) as “negative” social capital—patterns of connection that draw individuals into more high-risk situations [12]. In the latter case, risk-reducing connections such as those providing social support are balanced by other relationships with less salubrious outcomes [11]. In rural Puerto Rico, this relationship between having a high number of risk partners and lacking the means to obtain sufficient injection equipment seems to hold.

These findings support those of Kumar et al. [11] and provide further evidence of complex dynamics in the social capital among PWID. We note that paying for a syringe from a seller or receiving one free from the same source, as well as finding one found on the street, are the only syringe sources that do not require an additional, non-financial resource—payment does not require relationships implied by other categories such as friends, relatives, or even consistent “running partners” (i.e., consistent co-users) or access to locations beyond those necessary to obtain drugs. Ironically, in rural Puerto Rico, social resources necessary to access safe injection materials can be harder to obtain than simple cash. Access to syringe exchange programs and pharmacy-based sources necessitate a trip to a location, or in the case of a mobile needle exchange, a consistent pattern of location necessary to facilitate a meeting. Both of these require time, means for communication (such as a phone), and/or transportation. Receiving a syringe from a friend or relative requires having a relationship with a friend or relative who is willing to give you one (rather than, say, just giving you the $1 necessary to purchase one at the “punto” or drug purchase location). Receiving a needle for free from a seller indicates the presence of a relationship, but one that is clearly determined by the seller and not the PWID. In these ways, all of the options other than obtaining a needle from a seller (or finding one on the street—which is largely a matter of chance) require some form of reliable non-financial social capital.

What seems clear from the results shown here is that the forms of social capital necessary to obtain safe injection equipment may run counter to the sorts of relationships necessary to obtain other social resources, especially regular and reliable sources of drug sharing partners need to perform *caballo* [46]. For highly heroin-dependent users, the frequent practice of *caballo* normally requires maintaining a high number of co-injection relationships—being available to others in order to have them available to oneself—and finding a ready partner when one is in danger of withdrawal. The latter—a large network of potential *caballo* partners—is thus a form of social capital, but not one that is normally spoken of as such. Still, if social capital is the ability to access resources, we must be open to the idea that different resource “fields,” in the worlds of Pierre Bourdieu [62], may imply very different sorts of relationships. In this case, the social effort to stave off the physical effects of addiction seems to encourage relationships with highly risky implications, and which do not easily translate into relationships that promote access to safe injection equipment.

In this way, a high number of partners do not imply a lack of overall social capital but a lack of the sort of social capital necessary to obtain sufficient safe injection equipment. Highly networked/high-frequency users may continue to rely on a combination of sources to get the high volume of syringes needed for high rates of injection, and our results should not be read to indicate that highly networked individuals rely entirely on unsafe sources. Rather, our results show that only highly networked users show a statistically significant tendency to access high-risk sources, while less-networked users are largely undifferentiated in their use of other sources. Ethnographic research with this population shows that all users prefer to obtain needles from an SEP, where they can be guaranteed safe equipment that can be obtained for free.

A focus on social capital shows us that multiple forms of social capital are involved in the injection process, and that, contrary to general theoretical suggestions by Bourdieu [63] and practical suggestions by Lovell [61], different forms of social capital may not be convertible from one domain to another. Put another way, maintaining a high number of partners necessary for *caballo* may not contribute to having a high level of social support for obtaining safe syringes. For highly networked/high-frequency users, the sources of social capital necessary to support safe injection are clearly less than the social capital they maintain to perform *caballo*. Their inability to convert one form of capital to the other (at sufficient levels to inject safely) may elevate overall network risk of transmitting injection-related pathogens. We feel that such an understanding is more accurate than what is implied by “negative” social capital.

### Limitations

There are two key limitations to this study. First, we have a relatively small dataset, which limits our predictive power. Second, we lost as many as 43 respondents from the overall sample, over 10% of our dataset, to non-response on our dependent variables. However, some of this is mitigated by our focus on comparison between sources, as there were very few differences in missing data between the six sources. In addition, our study is limited in that it only asks if a respondent acquired a syringe from a given source in the last year. It may be beneficial to examine this type of question using a scale, whereby a measure of use frequency for each source is estimated by the respondents. Additional information on perceived risk associated with each source could provide insight into the actual conditions of the syringes received. Had we asked respondents about their preferences for sources, or the extent to which they ranked their various options, such data could help establish the difference between individual choice and overall social constraint on those choices. However, these needs were not anticipated prior to data collection, and we did not ask questions related to source preferences or a ranking of choices.

While our missing data would typically be a very important limitation, the goal of this paper is to draw comparisons between those who use each source, and there is little variation in missing data between sources. In other words, almost anyone who failed to answer questions about using a single source typically failed to answer about using all sources, meaning that comparisons across sources therefore drew on nearly identical samples. One result of this listwise deletion, however, is that differences found between those who use risky sources and those who do not are potentially underestimated; it is possible that the riskiest respondents were more likely to refuse to answer these questions. For this reason, we view the results of this analysis as a conservative estimate of differences between acquisition classes.

Additional limits include the fact that we have no way of knowing if any steps were taken prior to co-injection that would decrease the likelihood of disease risk (e.g., if syringes were cleaned prior to use). Taking steps like these decrease risks associated with co-injection in a way that is not measured when simply looking at the act of sharing. Second, including a measure of how frequent someone’s seller sells drugs could also potentially yield interesting information about the nature of the seller/user relationship through which the syringes associated with Table 5 were obtained, providing important information about this aspect of PWID social capital.

## Conclusion

Our results show limited association between how many injection partners someone has and whether or not they acquire needles from exchange programs, pharmacies, friends and relatives, from their sellers, or off the street. There is evidence of a positive relationship between obtaining a syringe from a seller, or off the street, and having more injection partners. Our interpretation of these findings centers on the fact that the latter are the only sources that do not require non-monetary resources, such as the sorts of social relationships implicit in accessing syringes from a friend, pharmacy, or syringe exchange. Obtaining a syringe from a seller only requires money, which is already required to purchase drugs and is co-located with that transaction. This suggests that facilitating the access of highly networked, high-frequency injectors to safer sources of syringes such pharmacies and syringe exchange providers can contribute to limiting risk practices among this group, and that current levels of secondary access via friends and co-injectors are insufficient to meet these needs.
